# Improved Viability of Spray-Dried *Pantoea agglomerans* for Phage-Carrier Mediated Control of Fire Blight

**DOI:** 10.3390/v16020257

**Published:** 2024-02-06

**Authors:** Nassereldin Ibrahim, Darlene Nesbitt, Qian (Tracy) Guo, Janet Lin, Antonet Svircev, Qi Wang, Joel T. Weadge, Hany Anany

**Affiliations:** 1 Agriculture and Agri-Food Canada (AAFC), Guelph Research and Development Centre (GRDC), 93 Stone Rd W., Guelph, ON N1G 5C9, Canada; nasser.ibrahim@agr.gc.ca (N.I.); qian.guo@agr.gc.ca (Q.G.); qi.wang2@agr.gc.ca (Q.W.); 2 Department of Biology, Wilfrid Laurier University, Waterloo, ON N2L 3C5, Canada; jweadge@wlu.ca; 3 Genetic Engineering and Biotechnology Research Institute, University of Sadat City, Sadat City 32897, Egypt; 4 Agriculture and Agri-Food Canada (AAFC), Vineland Station, ON L0R 2E0, Canada; darlene.nesbitt@agr.gc.ca (D.N.); antonet.svircev@agr.gc.ca (A.S.); 5 National Microbiology Laboratory, National Health Agency, 110 Stone Rd. W., Guelph, ON N1G 3W4, Canada; janet.t.lin@gmail.com; 6 Food Science Department, University of Guelph, Guelph, ON N1G 2W1, Canada

**Keywords:** biological control agent, *Erwinia amylovora*, antibiotic resistance, streptomycin, apple, pear

## Abstract

Fire blight, caused by *Erwinia amylovora*, is a devastating bacterial disease that threatens apple and pear production. It is mainly controlled by using antibiotics, such as streptomycin. Due to development of *E. amylovora* resistant strains and the excessive agricultural use of antibiotics, there is an increased awareness of the possibility of antibiotic resistance gene transfer to other microbes. Urgent development of biocontrol agents (BCAs) is needed that can be incorporated into integrated pest management programs as antibiotic alternatives. A novel phage-carrier system (PCS) that combines an antagonistic bacterium, *Pantoea agglomerans*, with its ability to act as a phage-carrier bacterium for *Erwinia* phages has been developed. The low viability of *P. agglomerans* cells following spray-drying (SD) has been a challenge for the industrial-scale production of this PCS. Here, an SD protocol was developed for *P. agglomerans* by modifying the growth medium and bacterial cell formulation using D(+)-trehalose and maltodextrin. The developed protocol is amenable to the industrial-scale production of the BCA/PCS. The *P. agglomerans* viability was greater than 90% after SD and had a shelf life at 4 °C of 4 months, and reconstituted cells showed a 3 log reduction in *E. amylovora* counts with a pear disc assay.

## 1. Introduction

Fire blight, caused by *Erwinia amylovora*, is a devastating bacterial disease that threatens apple and pear production worldwide [1]. Apple and pear infection by *E. amylovora* occurs during blossom opening, and the bacterial populations increase on the stigma. In the presence of dew droplets and rainwater, the bacterial cells flow down into the hypanthium and reach the natural flower openings. Bacterial cells then invade the plant tissue, resulting in the infection of blossoms and shoots [2]. Antibiotics, such as streptomycin and kasugamycin, have been noted to have a superior blossom blight control efficacy, with significant population reductions in *E. amylovora* on apple flower stigmas [3]. In Canada and the US, streptomycin and kasugamycin are the most commonly used pesticides against fire blight [4]. However, streptomycin-resistant strains of *E. amylovora* have been identified in different countries around the world [5,6,7]. This trend is concerning, as there is an increasing awareness of the possibility of antibiotic resistance gene transfer to human and animal pathogenic microbes due to the excessive use of antibiotics in agriculture [8,9,10,11]. Currently, the European Union regulatory agencies have restricted the use of streptomycin in agriculture, while Canada and the USA have only prohibited its use for organic farms [12].

Commercial biocontrol agents (BCAs) using antagonistic bacteria or lytic bacteriophages are available [8,13,14]. The bacterial BCA mode of action relies on competition for niches, nutrients and/or induction of plant defenses [15]. Bacterial-dependent BCAs against fire blight are commercially available, such as BlightBan^TM^ A506 (Nufarm, IL, USA) and Bloomtime Biological^TM^ (Verdesian Life Sciences LLC, Cary, NC, USA [16]. Lytic phage-dependent BCAs are also commercially available (e.g., AgriPhage–Fireblight by OmniLytics, West Sandy, UT, USA) and have been authorized by the US Environmental Protection Agency (EPA) for fire blight treatment [17,18,19]. The phage-carrier system (PCS) is a new form of BCA that combines antagonistic bacteria with phages as a double-pronged treatment that was developed for fire blight control [20,21,22,23]. The phages used in these treatments are able to infect both the antagonistic bacteria, *Pantoea agglomerans* (i.e., the phage carrier) and *E. amylovora* (i.e., the pathogen). A study using PCS with *P. agglomerans* and *E. amylovora* phages showed a 56% reduced infection on flowers of potted apple trees, and this treatment was comparable to the application of the antibiotic streptomycin [24]. Thus, PCS systems like this offer an opportunity to move away from using antibiotics and the resultant antibiotic resistance that develops in these agricultural sectors.

Spray-drying and freeze-drying are the most widely used methods for producing BCA powders. The spray-drying approach is preferable, as it is characterized by a high production rate and open production mode, as well as being easily scaled to industrial levels. Furthermore, the spray-drying operation time and production costs are 30–50 fold lower when compared to the costly and time-consuming freeze-drying approach [25,26]. However, a significant drawback of spray-drying is the loss of cell viability due to the use of high temperature and rapid dehydration during the spray-drying process.

Previous spray-drying trials with *P. agglomerans* showed low viability (e.g., 3–29% viability) after spray-drying due to the heat sensitivity of these cells [27]. The research presented herein describes an optimized formulation and protocol that leads to the improved viability of spray-dried *P. agglomerans*. Notably, the protocol demonstrated that greater than 90% viability remained following spray-drying for both the *P. agglomerans* cells alone and the same cells infected with *Erwinia* phages as a PCS. In follow-up experiments, a green fruit disc assay confirmed the high efficacy of the spray-dried *P. agglomerans* to control *E. amylovora* growth. Thus, the spray-drying protocol developed herein provides a viable avenue to make industrial-scale production of *P. agglomerans* as a BCA or PCS possible. Moreover, this protocol has broader implications for other antagonistic bacteria that are known to be sensitive to spray-drying, given the easily adaptable formulation and protocol.

## 2. Materials and Methods

### 2.1. Bacterial Isolates

All bacterial strains used in this study are listed in Table 1. Cultures were stored at −80 °C in Microbank cryobeads (Pro-Bank Diagnostics, Richmond Hill, ON, Canada). To prepare the working culture stock, one Microbank cryobead was mixed with one drop of PBS buffer (10 mM, pH 7.2) and plated on 2.3% (*w*/*v*) Difco^TM^ nutrient agar (NA) plates (BD, Sparks, MD, USA). The plates were incubated for 16–18 h at 27 °C and then stored at 4 °C for 1 to 2 wks. Working cultures were obtained from the initial cultures by streaking single colonies onto NA and incubating at 27 °C for 16–18 h.

### 2.2. Bacteriophages

The two lytic *E. amylovora* bacteriophages used in this study are ϕEa21-4 (Myovirus) and ϕEa46-1-A1 (Podovirus) as listed in Table 2. To propagate each phage, a bacterial host (Table 2) suspension was prepared by suspending 5–6 colonies in 3 mL of 0.8% (*w*/*v*) nutrient broth (NB) (BD, Sparks, MD, USA) to obtain an OD_600_ of ~0.6. Using a 250 mL baffled Erlenmeyer flask, 100 µL of the bacterial suspension was added to 75 mL of NB, which was then incubated at 27 °C with 150 rpm shaking (New Brunswick Innova^®^ 42, Eppendorf, Hamburg, Germany) for 3–4 h. A 100 µL aliquot of phage stock (5.0 × 10^9^ PFU/mL) was added and the mixture was incubated for 16–18 h at 27 °C with 150 rpm shaking. Following incubation, 1 mL of chloroform was added to the culture and incubated with shaking for 5 min. The bacterial culture was subjected to centrifugation at 8500× *g* at 4 °C for 15 min, the pellet discarded, and the supernatant filtered through a 0.22 µm Steriflip filter (Millipore, Burlington, MA, USA). The working phage stocks were stored at 4 °C in dark amber glass vials until needed and the titers were determined by using qPCR (Section 2.3) [23].

### 2.3. Quantitative PCR (qPCR)

Phage titers and *P. agglomerans* cell numbers were determined using qPCR with a protocol described by Gayder and coworkers (2019). Briefly, phage working solutions (or lysate) were treated with DNase I to remove non-encapsulated phage DNA prior to qPCR. DNase I treatments contained 10 µL of phage lysate, 1 µL of 2000 U/mL DNase I (NEB, Ipswich, MA, USA), 2 µL of 10× DNase I buffer (NEB, Ipswich, MA, USA), and 7 µL of sterile distilled water. The solution was incubated for 40 min at 37 °C and then 20 min at 80 °C to inactivate the DNase I. For qPCR, the 20 µL reactions consisted of 4 µL of 5× EVOlution Probe qPCR Mix (Montreal Biotech Inc., Montreal, QC, Canada), 200 nM of each primer (Table 3), 100 mM of the probe, and 2 µL of the template.

The reaction conditions began with 10 min at 95 °C, followed by 40 cycles of 10 s at 95 °C and 45 s at 54 °C. Dilutions of the standard plasmid, pTotalStdA (10^11^, 10^8^, and 10^5^ copies/mL), which contains amplicons for *E. amylovora* and *P. agglomerans*, as well as the phages ϕEa21-4 and ϕEa46-1-A1, were included in every run to generate a standard curve. A linearly fit standard curve of Ct value vs. copies/mL of the pTotalStdA was used to calculate the sample population quantities (genomes/mL). Populations of bacteria and phage were directly interpreted from this curve with the assumption that each cell or phage capsid contained a single genome. All qPCR quantification was performed with a Bio-Rad CFX96 qPCR System (Bio-Rad, Hercules, CA, USA).

### 2.4. Bacterial Counting by Platting

Bacterial cell cultures or reconstituted suspensions were serially diluted in PBS and a 100 µL volume of these dilutions was spread on NA and then incubated for 16–18 h at 27 °C. Bacterial colonies were enumerated, and the final CFU/mL was calculated accordingly by taking into account the dilution and plating factors. For all enumerations, the plating was carried out in at least triplicate.

### 2.5. Carrier Preparation

*P. agglomerans* was subcultured weekly on NA and, after 20 h at 27 °C, the plates were stored at 4 °C until needed that week. *P. agglomerans* cells were grown in NB as an initial rich-nutrient growth condition. For growth under stress conditions (i.e., osmotic-adaptation to increase the cell’s tolerance to heat stress and desiccation during spray-drying), *P. agglomerans* was grown on medium containing 5.0 g/L yeast extract, 10.0 g/L sucrose, and 35.5 g/L NaCl (YSN media) or in NB with the same amount of NaCl to reach a water activity (*a*_w_) in the medium of 0.98 *a*_w_ (where *a*_w_ is the ratio between the vapour pressure of a sample compared to a control) [27,29]. Cells were harvested by centrifugation (8500× *g* for 10 min at 4 °C), resuspended in 10 mM PBS at pH 7.2, and enumerated using the serial dilution and spread plating techniques on NA (outlined in Section 2.4). The colonies on the plates were counted after incubation for 16–18 h at 27 °C.

### 2.6. Phage-Carrier Formulation

#### 2.6.1. Material Screening and Formulation Optimization

Different concentrations and combinations of carbohydrates (all from Sigma-Aldrich, St. Louis, MO, USA) were used for formulations to explore with spray-drying (Table 4). These include D(+)-trehalose dihydrate, maltodextrin (dextrose equivalents 4.0–7.0 and 16.5–19.5), gum Arabic (from acacia tree), and carboxymethylcellulose sodium salt (CMC) with talc. *P. agglomerans* cell pellets (growth conditions mentioned in Section 2.5) were resuspended in a formulation mixture for 30 min to produce the formulated cells (f*Pa*).

The effect of different formulation chemicals on the *P. agglomerans*’ carrier infectivity by *Erwinia* phages was tested before selecting the suitable formulation for spray-drying. Different concentrations of formulation chemicals were prepared in NB, as outlined in Table 4, and inoculated with *P. agglomerans* Pa39-7 (10^3^ CFU/mL) together with the ϕEa21-4 phage (10^5^ PFU/mL) in 96-well plates prior to incubation at 27 °C in a Synergy H1 BioTek microplate reader (Agilent, Santa Clara, CA, USA) for 24 h. Readings of the plates at an OD_600_ were taken every 15 min and compared with the control sample (no phage added) in order to monitor growth/infectivity. After the initial trials, further optimization was carried out using different concentrations and combinations of the selected formulation chemicals as shown in Table 5.

#### 2.6.2. Spray-Drying of Phage-Infected and Non-Infected Carrier Cells

Osmotically stressed or non-stressed *P. agglomerans* strain Pa39-7 cells (s*Pa* or ns*Pa*, respectively) were prepared by mixing cell pellets with a formulation solution (1 g/100 mL) for 30 min before spray-drying to form formulated stressed (sf*Pa*) or formulated non-stressed (nsf*Pa*) samples. For phage-infected cells (i.e., the phage-carrier system), *P. agglomerans* cell pellets were resuspended in PBS and mixed with the phage (ϕEa21-4 or ϕEa46-1-A1) at an MOI of 1.0 for 30 min. The cells were then harvested by centrifugation (8500× *g* for 10 min at 4 °C) and resuspended in the formulation mixture (1 g/100 mL) for spray-drying. The spray-drying process was carried out using a Yamato ADL311S model (Yamato Scientific Co., Tokyo, Japan) with a feeding rate of 11–14 mL/min, inlet temperature of 160 °C, and an outlet temperature of 60 °C. The spray-dried powder was collected and stored in white plastic polyethylene high density tubes [30] at 4 °C or room temperature (RT) with activated Silica gel packets (5 g each) (Millipore Sigma, Oakville, ON, Canada).

### 2.7. Storage Stability of the Spray-Dried Preparations

Spray-dried powder of phage-infected and non-infected carrier cells was collected and stored in polyethylene tubes (as mentioned above), then enclosed in Statshield^®^ Moisture Barrier Al-bags (DESCO, Chino, CA, USA) and sealed with an Impulse Sealer, model AIE 300 (American International Electric, City of Industry, CA, USA). Spray-dried samples in sealed tubes were stored at RT or 4 °C for 16 wks (~4 months). At the time of sampling (biweekly), one tube was taken, and three technical replicates were prepared by reconstitution of the powder in sterile distilled water (0.9 g/3 mL). *P. agglomerans* cells were enumerated by plating on NA, and the logarithmic cell counts were plotted versus time. To test the presence of phage particles in the PCS powder and the capability of the phage to propagate (i.e., lyse the carrier cells) after reconstitution, 100 µL of reconstituted powder was used to inoculate 10 mL of NB and incubated at 27 °C with shaking at 180 rpm (New Brunswick Innova^®^ 42, Eppendorf, Hamburg, Germany). The phages were enumerated by qPCR (according to Section 2.3) at time intervals of 0, 2, 4, and 18 h (T_0_, T_2_, T_4_, and T_O/N_). The viability of the phage following spray-drying and reconstitution of the PCS powder was also tested by plating 100 μL of a 1/10 dilution of this resuspension on NA plates and incubating it for 16–18 h at 27 °C. These plates have a lawn of *Pantoea* growth from the reconstituted powder and any plaques on these plates were an indication that the phage (which had infected some of the *Pantoea*) were still viable. The PFU/mL was calculated after taking into account the dilution and plating factors.

### 2.8. Efficacy Testing of Phage-Carrier with a Green Pear Disc Assay

**Figure 1:**
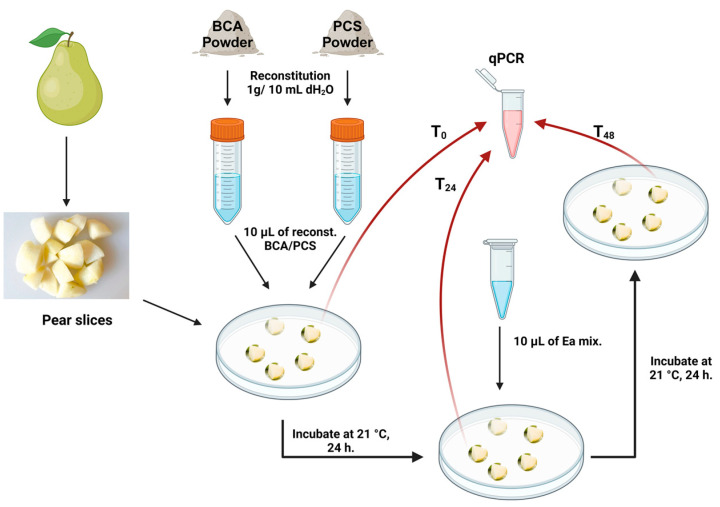
Testing BCA/PCS efficacy using a pear disc assay. Pear slices were inoculated with 10 μL of reconstituted BCA/PCS powder, separately, then incubated at 21 °C for 24 h. A mixture of the plant pathogens (*E. amylovora* strains Ea6-4 and EaD7, 1:1 mixture of 1 × 10^6^ CFU/mL each) was added and then incubated for an additional 24 h. At sampling times T_0_, T_24_, and T_48_, the pear disc was mixed with 500 μL buffer, sonicated for 3 min, and then 200 μL of this solution was heated at 80 °C for 10 min and then stored at −20 °C for qPCR analysis (red arrows).

A pathogenicity test was carried out using a green pear disc assay [31,32]. Briefly, green pears were purchased from a local grocery store, washed, rinsed, surface sterilized with 70% (*v*/*v*) ethanol, and rinsed twice with sterile distilled water. Under sterile conditions, a copper borer was used to cut into the fruit and produce pear plugs from which discs were cut measuring 1.5 mm in diameter and 1 mm in depth. The discs were placed into 2 mL sterile Eppendorf tubes using sterile forceps. Treatments consisted of formulated *P. agglomerans* Pa39-7, formulated *P. agglomerans* plus phages (strain Pa39-7 + phage ϕEa21-4), a positive control (1:1 ratio of *E. amylovora* strains *Ea*6-4 and *Ea*D7), and a negative control (non-infected formulated *P. agglomerans*). Two biological replicates, containing eight technical replicates were set up for each treatment. To each pear plug, 10 µL of treatment was added and sample tubes were collected at 0, 24, and 48 h. A 10 µL volume of pathogen was added at 10^6^ CFU/mL at 24 h and allowed to incubate for an additional 24 h. Following this time period, 500 µL of 0.01 M phosphate buffer pH 7.0 was added to the tubes containing the disc and sonicated for 3 min at high power for 3 min. A 200 µL volume of the sonicated supernatant was removed into 2 mL sterile Eppendorf tubes and the cells were heat-killed at 80 °C for 10 min (Figure 1). The heat-treated supernatant was frozen at −20 °C prior to evaluation by qPCR (Section 2.3). A Student’s *t*-test was performed comparing the means between the 24 and 48 h experimental data. Statistical analyses were performed using the JMP programme (JMP Statistical Discovery LLC, Cary, NC, USA). A three-way random analysis of variance (ANOVA) was performed on natural log (ln) + 1 transformed data comparing the effects of the two formulated products in suppressing the population of *E. amylovora* in vitro on green pear discs over time.

### 2.9. Powder Physical Properties

#### 2.9.1. Water Activity (*a*_w_)

Carrier and PCS powder water activities were measured by using an Aqualab 4TE water activity meter according to the manufacturer’s instructions [33]. Three technical replicates of powders were prepared and tested for the shelf-life experiment.

#### 2.9.2. Transmission Electron Microscopy (TEM)

Copper–rhodium (150-mesh) grids were covered with a thin layer of amorphous carbon made hydrophilic by a 45 s vacuum glow-discharge. PCS powder was rehydrated in filtered MilliQ water and then 4–6 μL was placed on individual grids and left for 2 min to adsorb. Excess sample was gently removed by filter paper. The grid was then washed three times with water. Sample on the grids were stained with 2% (*w*/*v*) uranyl acetate. A Tecnai G2 transmission electron microscope (FEI Company, Hillsboro, OR, USA) at the University of Guelph Electron Microscopy Unit (Guelph, ON, Canada) was used for visualization, operating at 200 kV under variable magnification (×50,000). Images were acquired using the Gatan Ultascan 4K CCD (Gatan Inc., Pleasanton, CA, USA) and Gatan Digital Micrograph software imaging system (version 3.5).

## 3. Results

### 3.1. Material Selection for the Phage-Carrier Formulation

**Figure 2:**
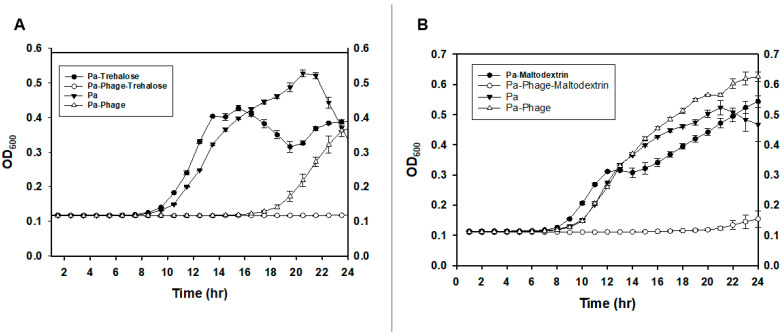
Determination of ϕEa21-4 phage infection of *P. agglomerans* cells in the presence of different formulation chemicals. (**A**) Survival of the *P. agglomerans* strain Pa39-7 after phage ϕEa21-4 infection in the presence (○) and absence (△) of D(+)-trehalose. Uninfected *P. agglomerans* Pa39-7 growth in the presence (•) and absence (▼) of D(+)-trehalose was also measured as controls. (**B**) Survival of the *P. agglomerans* strain Pa39-7 after phage ϕEa21-4 infection in the presence (○) and absence (△) of maltodextrin. In the absence of phage, *P. agglomerans* Pa39-7 growth in the presence (•) or absence (▼) of maltodextrin was similar. Assays were carried out in triplicate and the data averages and standard deviation were plotted. The final concentration of D(+)trehalose and maltodextrin was 5.0% each.

To exclude the possibility of interference of the formulation chemicals on the phage infectivity of the carrier cells after reconstitution, the lytic activity on *P. agglomerans* strain Pa39-7 cells by the ϕEa21-4 phage was assessed by turbidity measurements of cultures in the presence of each formulation chemical being tested. Phage infectivity and the growth of carrier cells was not significantly affected by the presence of D(+)-trehalose (Figure 2A) or maltodextrin (Figure 2B). Interestingly, the presence of D(+)-trehalose or maltodextrin actually augmented the ability of the phage to infect the host cells. Similarly, the presence of talc/CMC did not affect the growth of the carrier cells or their infectivity by ϕEa21-4 phage (Figures S1 and S2).

**Figure 3:**
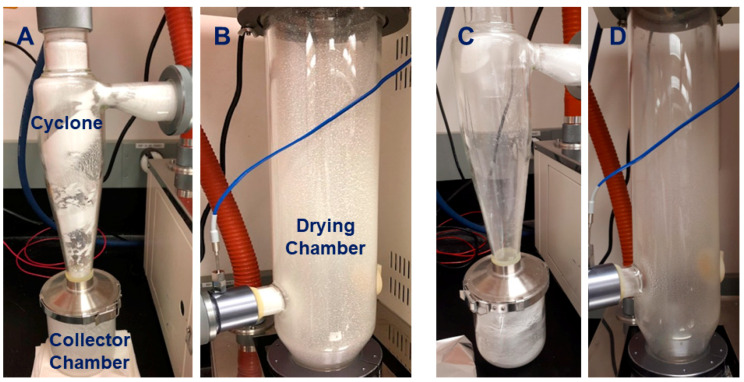
Visual representation of the spray-drying optimization process. One of the optimization criteria was the powder distribution during the spray-drying process. Using Formulation I (20% D(+)-trehalose and 2% talc/CMC), in addition to nozzle clogging by talc particles, collecting the powder was a challenge due to its adhesion to the drying chamber and the wall of the spray-dryer, as shown in (**A**,**B**). Using Formulation II (15% of D(+)-trehalose and 15% maltodextrin (16.5–19.5 dextrin equivalent) resulted in enhanced powder collection as shown in (**C**,**D**), as well as a reduced reconstitution time of 30 min instead of 3 h that was needed with the first formulation.

Based on the formulation screening results, 20% (*w*/*v*) D(+)-trehalose with 2% (*w*/*v*) talc and 0.1% (*w*/*v*) CMC was selected as Formula I for cell preparations before spray-drying. Despite the promising results, there were downstream application troubles using Formula I. Following reconstitution of the spray-dried powders for the biocontrol assay, the atomizer nozzle became clogged by the talc particles. Hence, further optimization was carried out using different mixtures of carbohydrates, as shown in Table 5, to solve the issue of nozzle clogging (Figure 3, Table S1). Accordingly, Formula II was a modified version containing 15% (*w*/*v*) of D(+)-trehalose and 15% (*w*/*v*) maltodextrin 16.5–19.5. This represented the final formulation that was used in all other experiments.

### 3.2. Optimization of Spray-Drying and Reconstitution Protocols

Several trials were carried out to determine the optimal spray-drying temperatures and reconstitution times of the spray-dried powders. Experiments were conducted at different inlet (150–160 °C) and outlet (50–60 °C) temperatures to assess the parameters that minimize the loss in viability of the carrier cells during the spray-drying process. According to this optimization, the best combination of inlet and outlet temperatures was determined to be 160 and 60 °C, respectively. The water activity of the powder (*a*_w_) was found to be 0.18 ± 0.002. After spray-drying, the powder produced was dissolved in sterile distilled water to 0.3 g/mL to retain the same weight/volume ratio used before the spray-drying process. The optimum reconstitution time was assessed by the powder solubility and cell plating on NA across a time course.

### 3.3. Confirmation of Cell Viability and Phage Infectivity after Spray-Drying

**Figure 4:**
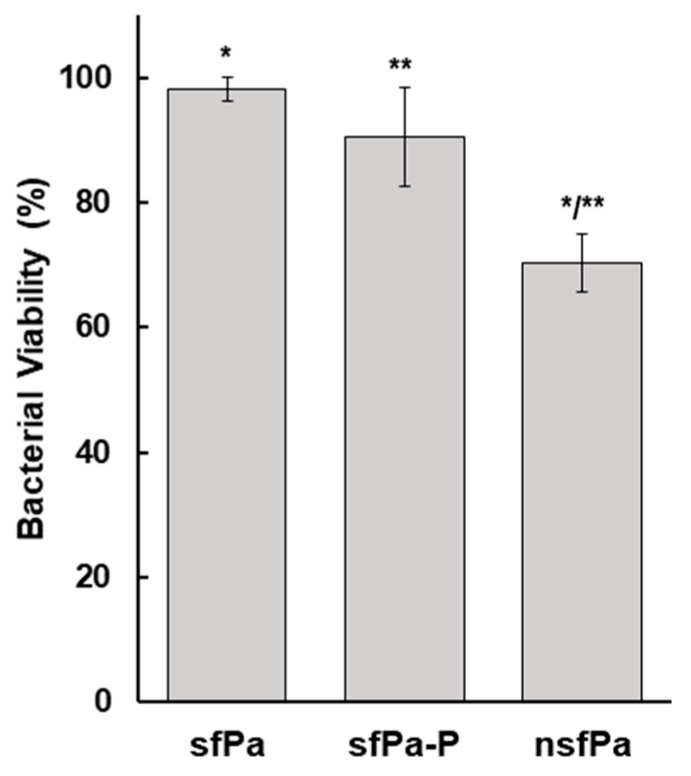
Viability of *P. agglomerans* after spray-drying. The viability of spray-dried *P. agglomerans* Pa39-7 was measured as a percentage by comparing before and after spray-drying cell counts. All tested *P. agglomerans* cells were formulated by the resuspension of cell pellets in Formula II. Both versions of the osmotically adapted cells that grew in NB plus NaCl medium (i.e., either phage-uninfected (sf*Pa*) or ϕEa21-4-phage-infected (sf*Pa*-P)), showed greater than 90% viability after spray-drying compared to osmotically non-adapted cells (nsf*Pa*), which showed about 70% cell viability. Assays were carried out in triplicate and the data averages and standard deviation were plotted. Using a *t*-test, the significance (*p* < 0.05) between sf*Pa* and nsf*Pa* (*), as well as between sf*Pa*-P and nsf*Pa* (**), was noted.

Once the formulation and spray-drying process was determined, the viability of formulated *P. agglomerans* carrier cells that grew in NB (nsf*Pa*) was compared to osmotically adapted cells grown in NB plus NaCl medium. The nsf*Pa* formulation displayed a 30% reduction in cell viability after spray-drying (Figure 4). However, osmotically adapted cells showed ≥95% of cell viability for carrier cells (sf*Pa*) and about 90% for the PCS infected carrier (sf*Pa*-P), as shown in Figure 4. The presented results are for the *P. agglomerans* strain Pa39-7 and ϕEa21-4 phage; however, the cell viability after spray-drying of the Pa39-7 strain infected with ϕEa46-1-A1 was similar to that infected with the ϕEa21-4 phage (Tables S2, S4 and S5). Also, the strain Pa31-4 showed a similar cell viability to Pa39-7 after spray-drying (Table S3).

**Figure 5:**
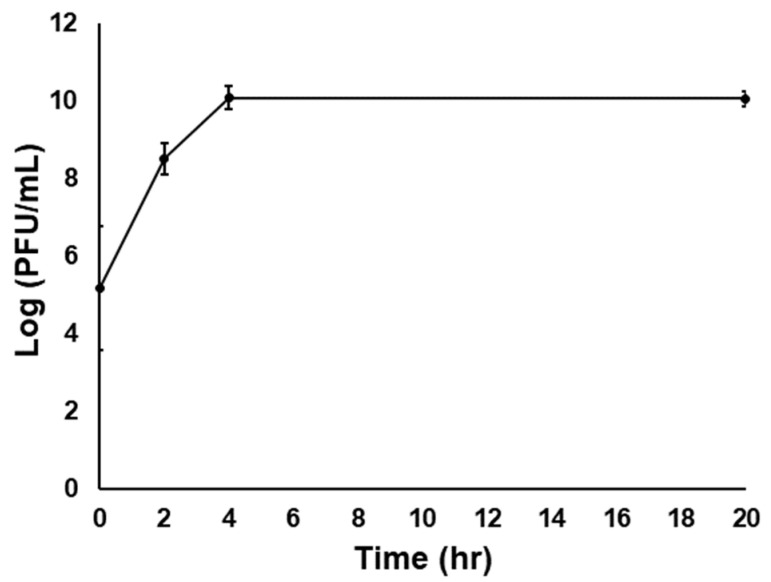
Infectivity of the ϕEa21-4 phage following reconstitution of the spray-dried powder. A volume of 100 μL of the reconstituted PCS powder (*P. agglomerans* Pa39-7 strain infected with ϕEa21-4) was used to inoculate 10 mL of NB and incubated at 27 °C with shaking at 180 rpm for 18 h before determining PFU/mL using qPCR. The graph displays the phage titer (log PFU/mL) increased with incubation time and that the phages were viable. Assays were carried out in triplicate and the data averages and standard deviation were plotted.

A 1/10 dilution of reconstituted PCS powder (i.e., from the sf*Pa*-P cells) was plated onto NA to mimic the double-layer plaque overlay assay to test the phage infectivity in the phage-carrier powder. After a 24 h incubation at 27 °C, plaques were observed, indicating the presence of active phage in the powder of spray-dried infected carrier cells. Furthermore, the phage titer increased by more than 4.5 log (PFU/mL) after 24 h when the reconstituted PCS powder was used to inoculate 10 mL of NB, as shown in Figure 5.

**Figure 6:**
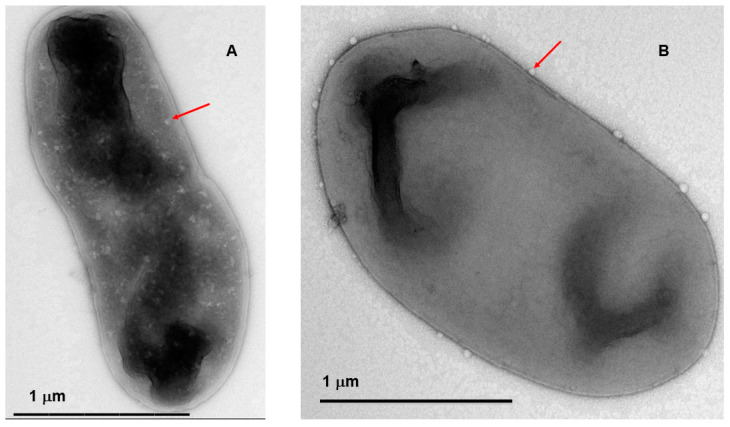
Electron microscopy of ϕEa21-4 after reconstitution of the spray-dried powder. Transmission electron microscopy images of the PCS reconstituted powder samples showed phage capsid (heads) (red arrows) assembled inside (**A**), or phage attached to *P. agglomerans* strain Pa39-7 cells (**B**).

TEM images were taken to compliment the *P. agglomerans* viability and phage plaque assays with reconstituted PCS powder. The images show assembled phage capsid (heads) (red arrows) inside and outside of the carrier cells (Figure 6A,B, respectively). Overall, these results further confirm that osmotic adaptation in combination with Formula II act to protect the phage and carrier cells during the spray-drying process so that they are present and viable after storage and reconstitution (Figure 5 and Figure 6).

### 3.4. Shelf-Life of the Carrier and Phage-Carrier Spray-Dried Powders

**Figure 7:**
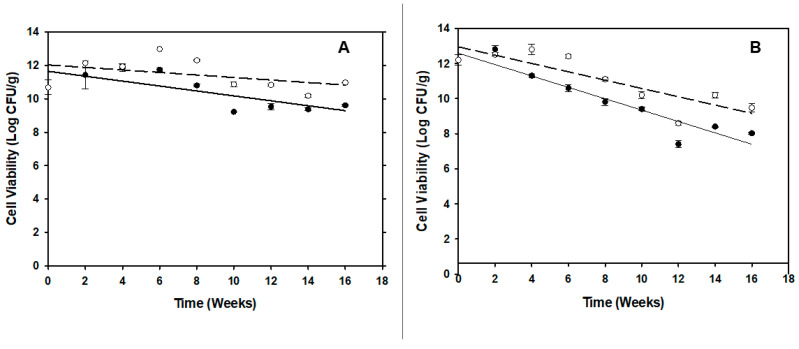
Changes in cell viability of *P. agglomerans* Pa39-7 cells in BCA or PCS powders after storage. (**A**) Changes in the cell viability of carrier cells in BCA powder stored at 4 °C (○) or at RT (•). (**B**) Changes in the cell viability of carrier cells in PCS powder stored at 4 °C (○) or at RT (•). Assays were carried out in triplicate and the data averages and standard deviation were plotted.

Cell count and phage infectivity assays were conducted every 2 wks for 4 months to assess the carrier and phage viability in the powder following storage. The viability of the *P. agglomerans* strain Pa39-7 in the carrier powder was reduced by more than 2 log (CFU/g) after 16 wks of storage at RT. However, only a 1–1.5 log reduction in bacterial counts was observed in powder stored at 4 °C (Figure 7A). For the PCS powder, *P. agglomerans* viability was reduced by 4 log CFU/g after 16 wks at RT and maintained an approximate 2.5 log reduction when stored at 4 °C (Figure 7B). Inoculation of reconstituted PCS powders in NB to test phage infectivity gave a similar pattern to that mentioned before in Figure 5.

### 3.5. Phage-Carrier Efficacy Assessment with the Pear Disc Assay

**Figure 8:**
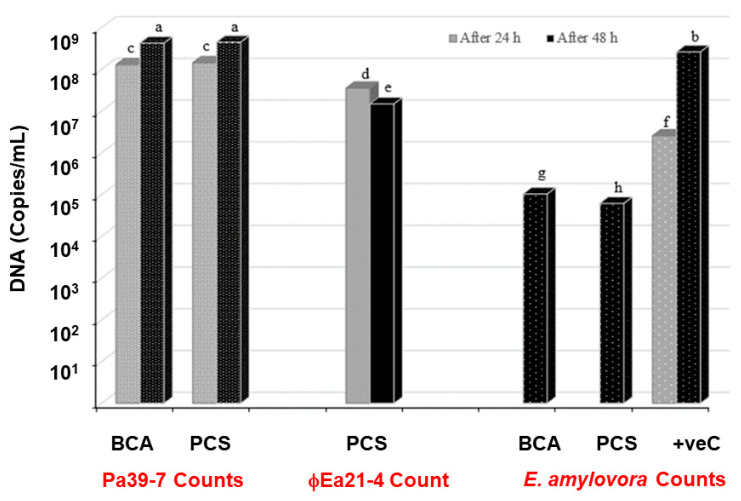
Populations of *P. agglomerans*, ϕEa21-4, and *E. amylovora* on green pear discs measured at 24 and 48 h. On the x-axis, the initial individual application to the pear discs is noted in black text, where the BCA (i.e., containing spray-dried *P. agglomerans* strain Pa39-7) and PCS (i.e., containing spray-dried ϕEa21-4 phage-infected *P. agglomerans* strain Pa39-7) formulations were tested and *E. amylovora* alone served as a positive control (+veC). The red text on the x-axis denotes what was enumerated by qPCR from the trial and is shown in the bar graph above. Grey bars represent populations on the pear discs at 24 h. For the statistically noted c and d samples, this is the bacterial count before inoculation with *E. amylovora*, while in the +veC (f) represents the *E. amylovora* numbers at the time of *E. amylovora* inoculations. All bars at 48 h (black) represent the growth of each organism in each formulation after 24 h of additional growth in the presence of *E. amylovora*. Statistical analyses showed that the interactions between all variable combinations in the experiment were highly significant (*p* < 0.0001), as denoted by the letters a–h above the bars. Replicate and random effects in the analysis were not significant (*p* > 0.05), indicating there was no variability between replicates of individual groups.

A pear disc was used as an in vitro assay to assess the protection that the BCA and PCS powder formulations can afford to plants when challenged with *E. amylovora* (Figure 1). In these trials, the BCA (only *P. agglomerans* strain Pa39-7) and PCS (*P. agglomerans* strain Pa39-7 and phage ϕEa21-4) were not significantly different from each other in population growth during the 24 h and 48 h sampling on the pear discs (Figure 8; c and a, respectively). Using this assay, *E. amylovora* was also noted to grow on the pear discs and increased significantly by 2 logs between the 24 h to 48 h time points (Figure 8; f and b, respectively). When the myovirus ϕEa21-4 population was monitored on the pear discs, a slight reduction in titer at 48 h after being exposed to *E. amylovora* for 24 h was noted (Figure 8; d to e statistical comparison). When the *E. amylovora* population was followed by qPCR, both the BCA (Pa39-7) and PCS (Pa39-7 + ϕEa21-4) led to significant reductions in *E. amylovora* numbers at the 48 h timepoint (Figure 8; g and h compared to b). Interestingly, the BCA was noted to have a slightly more significant effect in suppressing the *E. amylovora* pathogen on the green pear discs than PCS under these assay conditions (Figure 8; g and h, respectively).

## 4. Discussion

In fire blight management, BCAs and phages (registered in the USA) are commercially available as alternatives to antibiotic utilization [8,16,17]. Subsequently, PCS has been developed as an additional approach that combines the biological activity of antagonistic bacteria, *P. agglomerans*, and *Erwinia* phages that specifically target and kill the fire blight pathogen [20,21,34]. An optimized and economical production protocol is required for industrial-scale production of the PCS, and spray-drying or freeze-drying are the most widely used methods for production [25]. However, freeze-drying is costly, time-consuming, and operates as a closed system, which means that it cannot be used in a continuous large-scale production mode. In contrast, spray-drying has a higher powder production rate, continuous production mode, is easily scalable to industrial levels, and is 30–50-fold more cost effective compared to freeze-drying [25,26]. The major drawback of spray-drying is the loss of bacterial and/or phage viability due to exposure to high temperatures during preparation. For example, *P. agglomerans* carrier cells are thermally sensitive and have exhibited a significant reduction in cell viability (only 3–29% cell viability following spray-drying) [27,35,36]. Bacterial growth under osmotically adapted conditions is known to increase tolerance to desiccation. More specifically, osmotically adapted *P. agglomerans* can accumulate glycine-betaine, ectoine, and D(+)-trehalose [29,36,37]. Under this premise, osmotically adapted (i.e., grown in a standard medium with NaCl to a water activity of 0.97–0.98) and spray-dried *Pantoea* were able to consistently display cell viabilities around 30% [27]. However, these low viability numbers remain a hindrance to industrial-scale production.

This study established an improved *P. agglomerans* viability protocol by optimizing the formulation mixtures, spray-drying temperatures, nozzle clogging, and reconstitution times. The culmination of this work indicates that *P. agglomerans* (either under non-stressed or osmotically non-adapted conditions) treated with Formula II have an approximately 70% survival after spray-drying. Further optimization of this protocol was carried out by growing *P. agglomerans* Pa39-7 on NB with NaCl at an *a*_w_ of 0.98 for 18–20 h. This osmotic adaptation further improved cell viability to more than 95% for *P. agglomerans* alone (BCA) or more than 90% for PCS, which far exceeds the previously published results of ~30% for osmotically adapted [36] and 3% for non-adapted [27]. Thus, this enhanced protocol represents a very promising approach for industrial-scale production of *P. agglomerans* as a BCA or PCS application to control fire blight but also more widely as an avenue to explore for other bacteria recalcitrant to these processes.

For the PCS, the modified protocol was also assessed for the survival and infectivity of the phage in the carrier bacteria. In the presence of D(+)-trehalose and maltodextrin, the survival of *P. agglomerans* with the phage was markedly lower, thereby suggesting that not only had the phage ϕEa21-4 survived the spray-drying process, but that the presence of Formula II components enhanced its infectivity (Figure 2A,B). The presence of formulation compounds has been suggested in the literature to increase the viscosity and hence phage adsorption and/or can lead to changes in bacterial metabolism [38,39]. While plausible as a reason for the observed increase, further research would be needed to confirm these possibilities in our studies.

Maintaining the viability of the carrier and the ability of these cells to be infected with *E. amylovora* phages are important factors for a suitable PCS. In this work, Formula II was noted to protect both phage-infected and non-infected cells, as shown by a combination of plaque assays and NB inoculation with reconstituted powder, as well as electron microscopy imaging results (Figure 5 and Figure 6). In addition, the powder storage protocol used in this study utilized low O_2_-permeability tubes, silica gel packets to control humidity, and enclosure of the bottle in Statshield^®^ Moisture Barrier Al-bags, along with different storage temperatures. Combing these storage conditions led to the high cell viability of both carrier and PCS powders for almost 4 months (i.e., only 1–2 log reductions in viability over this period). By this process, the high humidity content in the powder, which can cause the crystallization of the amorphous trehalose matrix and decrease the cell viability [40,41], appears to have been mitigated. Our low temperature results are also consistent with other studies that demonstrated that the powder storage temperature has previously been noted to be an important factor that can affect the viability of the cells during storage due to membrane lipid oxidation [42,43]. Thus, by combining all of these factors we have been able to generate a PCS that has proven longevity that is amenable to production and delivery for field applications in future studies with expanded trials.

As an initial in vitro test of the efficacy of the BCA and PCS powders in mitigating *E. amylovora* infections, reconstituted powders were used on pear slices as a model system of infection. Both the BCA and PCS preparations were able to decrease *E. amylovora* growth by a 3 log reduction compared to untreated samples after 24 h of infection time with *E. amylovora* (Figure 8). While the pear disc assay represents initial analyses in plants, future work will build on this in a full workup with greenhouse and field trials (e.g., with potted apples and field-grown trees at the blossom stage).

Interestingly, the populations of carrier and phage (in the case of PCS) were not significantly different from each other over the entire 48 h experiment. These results are in contrast to our separate experiment where reconstituted PCS in NB led to a doubling of the phage counts after 24 h with shaking at 27 °C (Figure 5). It is plausible that there are alterations or delays in the infection process for the phage in the pear disc assay given that these environmental conditions are likely to be different than the rich NB media. Indeed, published data have noted that there are several factors that can contribute to discrepancies in phage counts between assays. For example, the bacterial carrier metabolic state can play a vital role in phage adsorption/infectivity and consequently phage propagation. For instance, the burst size of T4 phage is dependent on the cell state of the *E. coli* strain B23 [44]. The T4 burst size can vary between 1 and 40 progeny/cell depending on whether they are in the mid-log or stationary phase of growth, where the latter may produce only one phage per cell since the cells have entered a hibernation of scavenger response mode [45]. The cell’s metabolic state can also influence the sigma factors present within cells, which can differentially orchestrate the activity of the DNA-dependent RNA polymerase (RNAP) holoenzyme (Eσ) during the transcription process [46]. For example, *E. coli* contains six σ factors, including σ^70^ that is present during exponential growth and σ^S^ that is found during the stationary phase [46,47] and activated by various stress signals, including hyperosmolarity [48]. In the present spray-drying protocol, *P. agglomerans* was exposed to salt stress during osmotic adaptation and stationary-phase growth, which combined with further phyllosphere pressures/responses led to a slower/lower release of phages and concomitantly higher numbers of *P. agglomerans*. While the PCS and BCA both showed antagonistic behavior towards *E. amylovora*, the pear disc assay highlights the importance of assaying beyond the typical in vitro rich culture scenarios and provides a future avenue to explore observations over longer time periods, MOI concentrations, reconstitution variables, and more.

## 5. Conclusions

An improved viability protocol for spray-dried *P. agglomerans* as a BCA and PCS was established. This protocol can be used for industrial-scale production of a *P. agglomerans* BCA or PCS that is ready for greenhouse and field trials. Given the efficacy of this protocol, it is likely to also have broader applicability to other thermal-sensitive BCAs in development as an example of iterative optimization that can have drastically different yields and improved cell viability from 3–30% to over 90%.

## Figures and Tables

**Table 1:** Bacterial strains used for optimizing the spray-drying method.

Strain	GenBank Accession Number	Ref.
*Pantoea agglomerans*		
Pa39-7	JACSWZ000000000	[18]
Pa31-4	JACSXE000000000	[18]
*Erwinia amylovora*		
Ea6-4	JAAEVD000000000	[23]
EaD7	JAAEUT000000000	[23]

**Table 2:** *E. amylovora* phages used to generate a PCS.

Phage	Species	GenBank Accession Number	Pathogen Host	Ref.
ϕEa21-4	*Kolesnikvirus* Ea214	NC_011811.1	Ea6-4	[22]
ϕEa46-1-A1	-	NA	EaD7	[22]

**Table 3:** Primers and probes used for qPCR enumeration.

Name	Species	Amplicon Size (bp)	Sequence (5′-3′)	Reference
END37-F	ϕEa21-4	149	TTCAGCTTTAGCGGCTTCGAGA	[23]
END37-R			AGCAAGCCCTTGAGGTAATGGA	
END37-P			/56-ROXN/AGTCGGTACACCTGCAACGTCAAGAT/3IAbRQSp/	
STS3-F	ϕEa46-1-A1	96	GACAAACAAGAACGCGGCAACTGA	[28]
STS3-R			ATACCCAGCAAGGCGTCAACCTTA	
STS3-P			/56-FAM/AGATGAAGTAGGTTATCTTCACAGTGCCCT/3BHQ_1/	
Ea-Lsc-F	*E. amylovora*	105	CGCTAACAGCAGATCGCA	[24]
Ea-Lsc-R			AAATACGCGCACGACCAT	
Ea-Lsc-P			/5Cy5/CTGATAATCCGCAATTCCAGGATG/3IAbRQsp/	
Pa-Gnd-F	*P. agglomerans*	73	TGGATGAAGCAGCGAACA	[24]
Pa-Gnd-R			GACAGAGGTTCGCCGAGA	
Pa-Gnd-P			/5HEX/AAATGGACCAGCCAGAGCTCACTG/3BHQ_1/	

**Table 4:** Carbohydrates used for screening.

Chemicals	Final Concentration (%, *w*/*v*)
D(+)-Trehalose	0.05, 0.50, 5.00
Maltodextrin (DE * 4.0–7.0)	0.05, 0.50, 5.00
Maltodextrin (DE 16.5–19.5)	0.05, 0.50, 5.00
Talc/CMC **	0.01, 0.10, 1.00

* DE:Dextrose equivalents; ** CMC: Carboxymethyl cellulose.

**Table 5:** Concentration ranges used for optimization of the carrier formulation.

Chemicals	Final Concentration (%, *w*/*v*)
D(+)-Trehalose	13–15
Maltodextrin *	5–15
Talc	0–2
CMC **	0–1

* Dextrose equivalents 16.5–19.5; ** CMC: Carboxymethyl cellulose.

## Data Availability

Data are contained within the article. The original contributions presented in the study are included in the article/Supplementary Material, further inquiries can be directed to the corresponding author/s.

## Supplementary Materials

The following supporting information can be downloaded at: https://www.mdpi.com/article/10.3390/v16020257/s1, Figure S1: Determination of ϕEa21-4 phage infection of *P. agglomerans* strain Pa39-7 cells in the presence of different formulation chemicals; Figure S2: Determination of ϕEa21-4 phage infection of *P. agglomerans* strain Pa39-7 cells in the presence of different formulation chemicals; Table S1: Trails for optimizing the formula to overcome nozzle blockage of the PCA; Table S2: Spray Drying Results for *P. agglomerans* Strain Pa39-7 Infected with ϕEa46-1-A1; Table S3: Spray Drying Results for *P. agglomerans* Strain Pa31-4; Table S4: Phage Propagation After Reconstitution of the PCS Powder of *P. agglomerans* strain Pa39-7 Infected with ϕEa21-4; Table S5: Phage propagation after Reconstitution of PCS Powder of *P. agglomerans* strain Pa39-7 Infected with ϕEa46-1-A1.

## References

[1] Malnoy M., Martens S., Norelli J.L., Barny M.-A., Sundin G.W., Smits T.H.M., Duffy B. (2012). Fire blight: Applied genomic insights of the pathogen and host. Annu. Rev. Phytopathol..

[2] Thomson S. (2000). Epidemiology of Fire Blight.

[3] Slack S.M., Walters K.J., Outwater C.A., Sundin G.W. (2020). Effect of kasugamycin, oxytetracycline, and streptomycin on in-orchard population dynamics of *Erwinia amylovora* on apple flower stigmas. Plant Dis..

[4] McManus P.S., Stockwell V.O., Sundin G.W., Jones A.L. (2002). Antibiotic use in plant agriculture. Annu. Rev. Phytopathol..

[5] Parcey M., Gayder S., Morley-Senkler V., Bakkeren G., Úrbez-Torres J.R., Ali S., Castle A.J., Svircev A.M. (2020). Comparative genomic analysis of *Erwinia amylovora* reveals novel insights in phylogenetic arrangement, plasmid diversity, and streptomycin resistance. Genomics.

[6] Tancos K.A., Villani S., Kuehne S., Borejsza-Wysocka E., Breth D., Carol J., Aldwinckle H.S., Cox K.D. (2016). Prevalence of streptomycin-resistant *Erwinia amylovora* in new york apple orchards. Plant Dis..

[7] Russo N., Burr T., Breth D., Aldwinckle H. (2008). Isolation of streptomycin-resistant isolates of *Erwinia amylovora* in new york. Plant Dis..

[8] Svircev A.M., Roach D., Castle A. (2018). Framing the future with bacteriophages in agriculture. Viruses.

[9] Freeland G., Hettiarachchy N., Atungulu G.G., Apple J., Mukherjee S. (2023). Strategies to combat antimicrobial resistance from farm to table. Food Rev. Int..

[10] Tang K.W.K., Millar B.C., Moore J.E. (2023). Antimicrobial resistance (AMR). Br. J. Biomed. Sci..

[11] Sagar P., Azeem A., Banjara S.K., Veleri S. (2023). The role of food chain in antimicrobial resistance spread and One Health approach to reduce risks. Int. J. Food Microbiol..

[12] Phillips I. (2007). Withdrawal of growth-promoting antibiotics in Europe and its effects in relation to human health. Int. J. Antimicrob. Agents.

[13] Gildea L., Ayariga J.A., Robertson B.K. (2022). Bacteriophages as biocontrol agents in livestock food production. Microorganisms.

[14] Svircev A.M., Castle A.J., Lehman S.M. (2010). Bacteriophages for control of phytopathogens in food production systems. Bacteriophages in the Control of Food and Waterborne Pathogens.

[15] Dagher F., Olishevska S., Philion V., Zheng J., Déziel E. (2020). Development of a novel biological control agent targeting the phytopathogen *Erwinia amylovora*. Heliyon.

[16] Mikiciński A., Puławska J., Molzhigitova A., Sobiczewski P. (2020). Bacterial species recognized for the first time for its biocontrol activity against fire blight (*Erwinia amylovora*). Eur. J. Plant Pathol..

[17] Wagemans J., Holtappels D., Vainio E., Rabiey M., Marzachì C., Herrero S., Ravanbakhsh M., Tebbe C.C., Ogliastro M., Ayllón M.A. (2022). Going viral: Virus-based biological control agents for plant protection. Annu. Rev. Phytopathol..

[18] Balogh B., Jones J.B., Momol M., Olson S., Obradovic A., King P., Jackson L. (2003). Improved efficacy of newly formulated bacteriophages for management of bacterial spot on tomato. Plant Dis..

[19] Flaherty J.E., Somodi G.C., Jones J.B., Harbaugh B.K., Jackson L.E. (2000). Control of bacterial spot on tomato in the greenhouse and field with h-mutant bacteriophages. HortScience.

[20] Boulé J., Sholberg P.L., Lehman S.M., O’Gorman D.T., Svircev A.M. (2011). Isolation and characterization of eight bacteriophages infecting *Erwinia amylovora* and their potential as biological control agents in British Columbia, Canada. Can. J. Plant Pathol..

[21] Gayder S., Parcey M., Nesbitt D., Castle A.J., Svircev A.M. (2020). Population dynamics between *Erwinia amylovora*, *Pantoea agglomerans* and bacteriophages: Exploiting synergy and competition to improve phage cocktail efficacy. Microorganisms.

[22] Gill J.J., Svircev A.M., Smith R., Castle A.J. (2003). Bacteriophages of *Erwinia amylovora*. Appl. Environ. Microbiol..

[23] Gayder S., Parcey M., Castle A.J., Svircev A.M. (2019). Host range of bacteriophages against a world-wide collection of *Erwinia amylovora* determined using a quantitative pcr assay. Viruses.

[24] Lehman S.M. (2007). Development of a Bacteriophage-Based Biopesticide for Fire Blight.

[25] Desobry S.A., Netto F.M., Labuza T.P. (1997). Comparison of spray-drying, drum-drying and freeze-drying for β-carotene encapsulation and preservation. J. Food Sci..

[26] Lian W.-C., Hsiao H.-C., Chou C.-C. (2002). Survival of bifidobacteria after spray-drying. Int. J. Food Microbiol..

[27] Costa E., Teixidó N., Usall J., Fons E., Gimeno V., Delgado J., Viñas I. (2002). Survival of pantoea agglomerans strain cpa-2 in a spray-drying process. J. Food Prot..

[28] Roach D.R. (2012). *Erwinia amylovora* Bacteriophage Resistance. Ph.D. Thesis.

[29] Teixidó N., Cañamás T.P., Usall J., Torres R., Magan N., Viñas I. (2005). Accumulation of the compatible solutes, glycine-betaine and ectoine, in osmotic stress adaptation and heat shock cross-protection in the biocontrol agent *Pantoea agglomerans* CPA-2. Lett. Appl. Microbiol..

[30] Torres R., Solsona C., Viñas I., Usall J., Plaza P., Teixidó N. (2014). Optimization of packaging and storage conditions of a freeze-dried *Pantoea agglomerans* formulation for controlling postharvest diseases in fruit. J. Appl. Microbiol..

[31] Aldwinckle H., Preczewski J. (1976). Susceptibility of vegetative tissues of apple cultivars to invasion by *Erwinia amylovora*. Phytopathology.

[32] Bogdanove A.J., Kim J.F., Wei Z., Kolchinsky P., Charkowski A.O., Conlin A.K., Collmer A., Beer S.V. (1998). Homology and functional similarity of an hrp-linked pathogenicity locus, dspEF, of *Erwinia amylovora* and the avirulence locus avrE of *Pseudomonas syringae* pathovar tomato. Proc. Natl. Acad. Sci. USA.

[33] Yu X., Schmidt A.R., Schmidt S.J. (2009). Uncertainty analysis of hygrometer-obtained water activity measurements of saturated salt slurries and food materials. Food Chem..

[34] Svircev A.M., Lehman S.M., Kim W.S., Barszcz E., Schneider K.E., Castle A.J. (2006). Control of the Fire Blight Pathogen with Bacteriophages.

[35] Teixidó N., Usall J., Torres R. (2022). Insight into a successful development of biocontrol agents: Production, formulation, packaging, and shelf life as key aspects. Horticulturae.

[36] Teixidó N., Cañamás T.P., Abadias M., Usall J., Solsona C., Casals C., Viñas I. (2006). Improving low water activity and desiccation tolerance of the biocontrol agent *Pantoea agglomerans* cpa-2 by osmotic treatments. J. Appl. Microbiol..

[37] Bonaterra A., Camps J., Montesinos E. (2005). Osmotically induced trehalose and glycine betaine accumulation improves tolerance to desiccation, survival and efficacy of the postharvest biocontrol agent *Pantoea agglomerans* EPS125. FEMS Microbiol. Lett..

[38] Manbua N., Suteewong T., Sae-Ueng U. (2022). Efficacy of sugar excipients on lyophilized c22 phage infectivity evaluated by atomic force microscopy. Biol. Control.

[39] Howes W.V. (1965). Effect of glucose on the capacity of *Escherichia coli* to be infected by a virulent lamba bacteriophage. J. Bacteriol..

[40] Vandenheuvel D., Meeus J., Lavigne R., Van den Mooter G. (2014). Instability of bacteriophages in spray-dried trehalose powders is caused by crystallization of the matrix. Int. J. Pharm..

[41] Moreira M.T.C., Martins E., Perrone Í.T., de Freitas R., Queiroz L.S., de Carvalho A.F. (2021). Challenges associated with spray drying of lactic acid bacteria: Understanding cell viability loss. Compr. Rev. Food Sci. Food Saf..

[42] Silva J., Freixo R., Gibbs P., Teixeira P. (2011). Spray-drying for the production of dried cultures. Int. J. Dairy Technol..

[43] Teixeira P., Castro H., Kirby R. (1996). Evidence of membrane lipid oxidation of spray-dried *Lactobacillus bulgaricus* during storage. Lett. Appl. Microbiol..

[44] Choi C., Kuatsjah E., Wu E., Yuan S. (2010). The effect of cell size on the burst size of t4 bacteriophageinfections of *Escherichia coli* b23. J. Exper. Microbiol. Immunol..

[45] Bryan D., El-Shibiny A., Hobbs Z., Porter J., Kutter E.M. (2016). Bacteriophage t4 infection of stationary phase *E. coli*: Life after log from a phage perspective. Front. Microbiol..

[46] Gross C.A., Chan C., Dombroski A., Gruber T., Sharp M., Tupy J., Young B. (1998). The functional and regulatory roles of sigma factors in transcription. Cold Spring Harb. Symp. Quant. Biol..

[47] Gruber T.M., Gross C.A. (2003). Multiple sigma subunits and the partitioning of bacterial transcription space. Annu. Rev. Microbiol..

[48] Hengge-Aronis R. (2002). Signal transduction and regulatory mechanisms involved in control of the sigma(s) (rpos) subunit of rna polymerase. Microbiol. Mol. Biol. Rev..

