# Supplementary material for: Improved Viability of Spray-Dried Pantoea agglomerans for Phage-Carrier Mediated Control of Fire Blight
Source: Viruses. 2024 Feb 6;16(2):257. doi: 10.3390/v16020257 (PMC10893313; doi:10.3390/v16020257)
Supplement: Supplementary file 1 [file viruses-16-00257-s001.zip › viruses-2817307-supplementary.pdf]

Supplemental Data

A.1 Polymer Selection for the Phage-Carrier Formulation

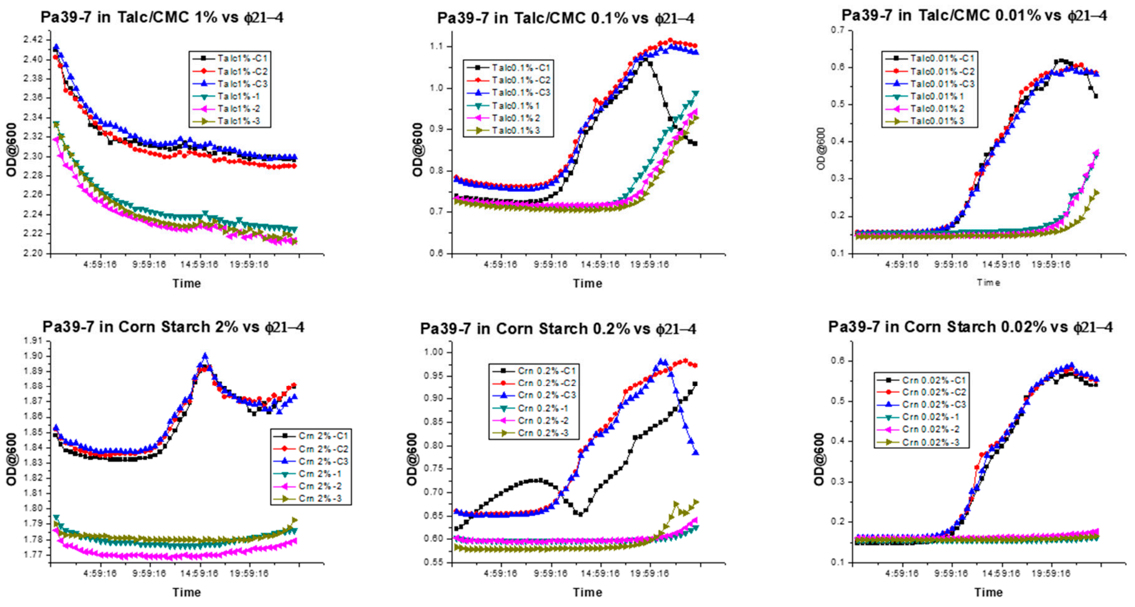

Figure S1. Determination of ϕEa21-4 phage infection of *P. agglomerans* strain Pa39-7 cells in the presence of different formulation chemicals. Survival of *P. agglomerans* Pa39-7 strain after phage ϕEa21-4 infection in the presence of Talc/CMC (Upper) and corn starch (Bottom). Assays were carried out in triplicate and the data averages and standard deviation were plotted.

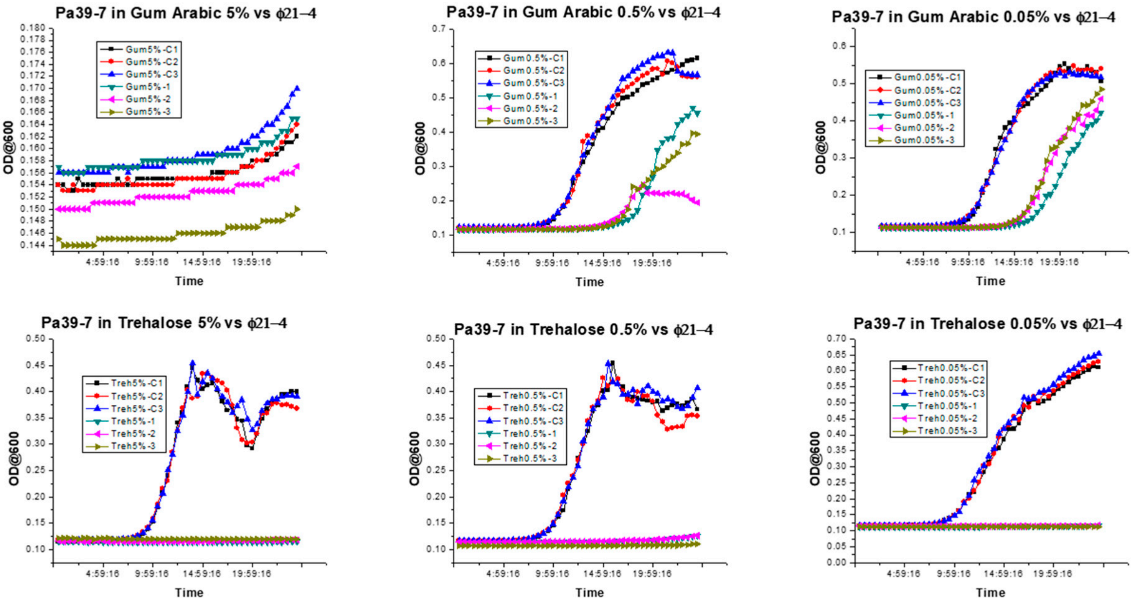

Figure S2. Determination of ϕEa21-4 phage infection of *P. agglomerans* strain Pa39-7 cells in the presence of different formulation chemicals. Survival of *P. agglomerans* Pa39-7 strain after phage ϕEa21-4 infection in the presence of gum arabic (Upper) and D(+)-trehalose (Bottom). Assays were carried out in triplicate and the data averages and standard deviation were plotted.

A.2 Formulation Optimization

Table S1: Trails for optimizing the formula to overcome nozzle blockage of the PCA.

Trials	Trehalose (%)	Maltodextrin (%)	Talc (%)	CMC (%)	Powder Recovery (%)	Notes	Bacterial log reduction after SD (CFU/mL)
No bacterial cells were added to the formula							
Trail 1	15	12.5	2	0.4	21.4		NA*
Trail 2	13	5.58	0	0.87	22.3		NA
Trail 3	15	12.5	0	0.4	24.8		NA
Trail 4	15	15	0	0.4	24.2		NA
Trail 5	15	15	0	0.4	27.6		NA
Trail 6	15	15	0	0.4	27.6		NA
Trail 7	15	18	0	0.4	26.9		NA
Pantoea cells (infected and not infected by phage) were added to the formula							
Trail 8	15	15	0	0.4	20.1		0.65
Trail 9	15	15	0	1	NA	Formulation too thick	1.15
Trail 10	15	15	0	0.8	NA	Formulation too thick	0.27
Trail 11	15	15	0	0.6	25.5	Powder is a little bit sticky.	ND**
Trail 12	15	15	0	0.4	2.8	The powder was sticky.	ND
Trail 13	15	15	0	0	26.8	Bacteria added to formulation	0.83
Trail 14	15	15	0	0	26.1	Bacteria added to formulation	0.54
Trail 15	15	15	0	0	24.1	Bacteria added to formulation	0.51
Trail 16	15	15	0	0	24.7	Bacteria and phages added to formulation	0.28
Trail 18	15	15	0	0	23.6	Bacteria and phages added to formulation	1.35
Trail 19	15	15	0	0	26.3	Bacteria and phages added to formulation	0.65

*NA: Not applicable; **ND : Not determined

Table S2: Spray Drying Results for *P. agglomerans* Strain Pa39-7 Infected with ϕEa46-1-A1

Pa39-7 infected with ϕEa46-1-A1*	Log CFU/mL
Before Spray drying	7.7
After Spray drying	6.6
Viability Reduction	1.1

* The survival viability after spray drying is similar to that with Pa39-7 infected with ϕEa21-4 phage (~ 1.0 log reduction). The survival viability is 87%.

Table S3: Spray Drying Results for *P. agglomerans* Strain Pa31-4*

Treatment	Before SD (CFU/mL)	After SD (CFU/mL)	Log reduction
Pa 31-4	$1.85x10^9$	$5.25x10^8$	0.55

* The survival viability after spray drying is similar to that with Pa39-7 (0.5-0.7 log reduction). The survival viability is 90%.

Table S4: Phage Propagation After Reconstitution of the PCS Powder of *P. agglomerans* strain Pa39-7 Infected with ϕEa21-4*

Time (Hrs)	Formulated**		Spray Dried**	
	Pa39-7	ϕEa21-4	Pa39-7	ϕEa21-4
T_0	8.8	5.7	8.7	7.1
T_2	9.0	8.8	8.9	7.7
T_4	8.7	9.7	8.8	8.8
T_6	9.3	10.0	9.0	9.2
T_{24}	9.6	10.0	9.6	9.4

* Inoculation of 10 mL NB with reconstituted powder of Pa39-7 infected with ϕEa21-4.

**Log Starting Quantity

Table S5: Phage propagation After Reconstitution of PCS Powder of *P. agglomerans* strain Pa39-7 Infected with ϕEa46-1-A1*

Time (Hrs)	Formulated**		Spray Dried**	
	Pa39-7	ϕEa46-1-A1	Pa39-7	ϕEa46-1-A1
T_0	9.0	6.2	8.7	5.7
T_2	9.1	8.2	8.8	5.9
T_4	9.2	9.6	8.8	5.7
T_6	9.6	9.8	9.0	7.0
T_{24}	9.5	9.5	9.4	9.6

* Inoculation of 10 mL NB with reconstituted powder of Pa39-7 infected with ϕEa46-A1.

**Log Starting Quantity
